# Body composition and grip strength constraints in elite male rink-hockey players of contrasting ethnicity

**DOI:** 10.1371/journal.pone.0274894

**Published:** 2022-09-22

**Authors:** António Ferraz, João Valente-Dos-Santos, Pedro Duarte-Mendes, Célia Nunes, Samuel Victorino, Manuel J. Coelho-e-Silva, Bruno Travassos

**Affiliations:** 1 CIFD, Sports Research and Training Center, Jean Piaget University of Angola, Luanda, Angola; 2 CIDESD, Research Center in Sports Sciences, Health Sciences and Human Development, Department of Sport Sciences, University of Beira Interior, Covilhã, Portugal; 3 Faculty of Physical Education and Sport, Lusófona University, Lisbon, Portugal; 4 University of Coimbra, FCDEF Coimbra, Coimbra, Portugal; 5 University of Coimbra, CIDAF Coimbra, Coimbra, Portugal; 6 Department of Sport and Well Being, Polytechnic Institute of Castelo Branco, Castelo Branco, Portugal; 7 Sport, Health and Exercise Research Unit—SHERU, Polytechnic Institute of Castelo Branco, Castelo Branco, Portugal; 8 Department of Mathematics and Center of Mathematics and Applications, University of Beira Interior, Covilhã, Portugal; Universiti Malaysia Terengganu, MALAYSIA

## Abstract

Rink hockey is a highly specialized and physiological demanding sport with sparse research regarding the game and athletes’ characteristics. A cross-sectional study was developed to characterize the body composition and grip strength of elite male rink hockey players and to establish the relationship between ethnicity on body composition and grip strength. A sample of 100 elite rink-hockey athletes aged 26.59 ± 6.02 participated in the study, comprised of 69 Caucasian male adults aged 27.58 ± 6.44 years and 31 Black African male adults aged 24.39 ± 4.27. Body composition was assessed by anthropometric measurements. Static grip strength was assessed with an adjustable dynamometer. Multiple regression analysis was applied to understand which variables constraints body fat percentage (BF) and grip strength. Body mass showed an average of 76.36 ± 9.18 kg for 175.80 ± 5.87 cm of height and BF% of 10.82 ± 5.07%. Maximal right grip strength was 50.91 ± 6.26 kg and 50.27 ± 6.23 kg for left grip strength. Four predictors accounted for 70.01% of the variance of BF%: abdominal circumference (*p* < 0.001), right thigh circumference (*p* < 0.001), right calf circumference (*p* = 0.001) and ethnicity (*p* = 0.016). Three predictors accounted for 13.1% of the variance of right grip strength: ethnicity (*p* = 0.013), chronological age (*p* = 0.024) and right distal thigh circumference (*p* = 0.014). Results suggest that elite rink hockey athletes have a specific anthropometric identity, which at the elite level may lead to reduced body fat and greater handgrip strength. Ethnicity seems to predict body fat and grip strength in elite rink hockey athletes.

## Introduction

Rink hockey is an indoor intermittent team sport that requires both short- and long-duration efforts, followed by incomplete recovery periods [1,2], that require highly specialized and physiological demands [3]. Players’ performance is highly dependent on different interacting factors such as health status, genetic endowment, physical training, fitness levels and body composition [4]. In line with previous studies, there are similarities between rink hockey and other hockey modalities such as ice hockey: (1) high-intensity intermittent skating and physical contact requiring a well-developed metabolism for short and long-duration efforts; and (2) multidirectional skating and the need to continually perform complex skills while gripping a stick [3,5].

It is known that the body composition characteristics of athletes constrain their competitive performance as well as their training, which is especially relevant for sports professionals [6–8]. Anthropometry is defined as the study of body dimensions and composition and is among the biological variables related to sports performance [9]. In the last decades, several researchers in different sports have focused their attention on the anthropometric characteristics of elite athletes [10,11] aiming to identify key determinants that characterize specific sports and/or specific positional groups of players [1]. Therefore, the analysis of an athlete’s anthropometry is an important tool to characterize and improve sports performance [1].

Grip strength is an important variable used to measure muscular strength, which is a vital component of physical fitness [12], as it can be used to gain relevant insights regarding bone health [13] and functional status [14]. Upper strength assessment has been shown to correlate to an individual’s general muscular strength [15], and the hand-grip strength test seems to be influenced by the level of physical activity and training [16]. Grip strength is mandatory in most hockey-specific movements with players continually performing complex skills while gripping a stick [5]. According to Coelho-e-Silva et al. [17] under-17 international male rink hockey players revealed particular body composition characteristics and high hand-grip strength when compared with local players, arguing that players with high hand-grip strength have a greater propensity towards becoming elite athletes.

Regarding ice hockey, a previous study emphasized that the grip hand is influenced by age [5]. Adult athletes revealed higher values (right = 67.34 ± 7.75kg; left = 67.03 ± 8.16kg) when compared to younger athletes [18]. However, there is no information or standard references regarding this relationship in adult elite athletes in this sport. To the best of our knowledge, the existing research regarding body composition with adult elite athletes was only assessed with small sample sizes. Furthermore, anyone has been conducted exploring normative grip strength performance in adult elite male rink-hockey players.

Fitness testing for monitoring and evaluating training adaptations is important for assessing the level of physical performance in athletes [19]. According to previous research, ethnicity also seems to be an important determinant factor for functional, morphological, and physiological adaptations such as strength, body fat and cardiovascular adaptations [20]. In the past few years, a relevant number of African athletes playing abroad prompted researchers to investigate the effect of exercise and sports training on those ethnicities. Generally, several researchers have shown that ethnic differences in body proportions can have a significant influence on the estimation of BF% derived using field methods [21]. Some authors suggest that the biological differences in the body composition between Black Africans and Caucasians may necessitate the need to develop prediction equations based on the specific population [21, 22]. However, to our best knowledge, there is no information regarding this characterization of Black elite male rink hockey players.

Therefore, the aim of the present research was to characterize and underline possible differences in body composition and grip strength between Black African and Caucasian ethnicities. Particularly it was our intention to identify the variables that constrain body composition and grip strength values according to ethnicity.

## Material and methods

### Ethics statement

Data collection was carried out according to the international ethical standards with humans [23] after approval by the Ethics Committee of the Universidade da Beira Interior (CE-UBI-Pj-2019-053:ID1519). All participants were informed about the aims, the protocol and procedures, and signed written informed consent. Participation was voluntary and each participant could withdraw at any time. Data was measured in Angola and Portugal between December 2019 and the end of February 2020.

### Sample

One hundred (100) elite rink-hockey athletes aged 26.59 ± 6.02 participated in the study. The sample comprised 69 Caucasian elite male rink-hockey players aged 27.58 ± 6.44 years, from the Portuguese first league, which was comprised of six (6) teams and six (6) nationalities (Portuguese n = 47; Spanish n = 11; Argentines n = 7; French n = 2; Italian n = 1; English n = 1) and 31 Black African elite male rink-hockey players aged 24.39 ± 4,27 from the Angolan first league, consisting of three (3) teams and (1) nationality (Angolan n = 31). The selection process of the sample was convenient considering the level of the clubs, and their availability to participate in this study. The inclusion criteria of the players were as follows: (1) must be between 18 and 43 years old, (2) must be continuously competing for at least 5 full years without having played any other sport in the past, and (3) attend at least four weekly training sessions in clubs registered at Federação Portuguesa de Patinagem and Federação Angolana de Patinagem [24].

### Anthropometric measurements

Height was measured with no shoes using a stadiometer (Seca 700: Seca, Hamburg Germany) to the nearest 0.1 cm. Body weight was measured in light clothing with no shoes using the Tanita (RD-953) digital weight scale. All skinfolds and circumferences were measured using an anthropometric tape (Harpenden, Holtain LTD, Germany) to the nearest 0.1cm [25] while the participants were in a standing position. Waist circumference was measured midway between the lower costal margin and the iliac crest and at minimal respiration. Hip girth circumference was measured round the buttocks at the level of the greatest trochanteric projections. Thigh girth circumference was measured at the highest tight position, while mid-thigh girth circumference was measured midway between the hip level and the knee. Lower-thigh girth circumference was measured 5cm from the top of the patella, and calf girth circunference was measured with legs slightly apart with body mass equally distributed on both legs. All circumferences assessed were as follow: *(1)* WC (Waist Circumference); *(2)* ABC (Abdominal Circumference); *(3)* HC (Hip Circumference); *(4)* RTC (Right Thigh Circumference); *(5)* RMTC; *(6)* Right mid-thigh Circumference); *(7)* RDTC (Right distal-tight Circumference); *(8)* RCC (Right Calf Circumference); *(9)* LTC (Left Tight Circumference); *(10)* LMTC (Left mid-tight Circumference); *(11)* LDTC (Left distal-tight Circumference); *(12)* LCC (Left Calf Circumference); Body fat percentage was derived from the three skinfolds Body Density (BD) formula of Jackson and Pollock [26], additionally, the BF% was calculated by using the Siri [27] formula. The thickness of the nine skinfolds (*triceps*, *subscapular*, *biceps*, *supra iliac*, *abdominal*, *pectoral*, *medium axillar*, *front-thigh*, and *media calf*) was measured using a Harpenden skinfold calliper (British Indicators, Burgess Hill, UK) and calculated according to Durnin and Womersley [28]. The summary of skinfolds (SUMSK) was also calculated. Body surface area (BSA) was derived from the Dubois formula [29].

### Reliability of anthropometric measurements

All measurements were taken by an experienced observer during both competition seasons. Body Mass Index (BMI) was calculated as [weight (kg)]/ [height (m)]^2^ and was classified according to WHO standards. To calculate the precision of measurements, intra-observer technical error of measurement (TEM) was calculated for skinfolds and circumferences (Note. TEM = PD2/2N, where D is the difference between pre- and post-measures and N is the sample size, and TEM% = 100 * TEM/X, where X is the grand mean of the pre- and post-measures) [30]. Ten rink hockey athletes were measured by the same observer. Skinfolds and circumferences were registered twice to calculate deviations between both measures. The mean TEM% for the 9 skinfolds was 5.8% and 1.8% for the 11 circumferences. The absolute TEM was acceptable for both measurements [30].

### Handgrip upper static strength

Grip strength was conducted to assess musculoskeletal fitness. Athletes used a handgrip dynamometer (T.K.K. 5401) adjustable to the hand size by an experienced observer. Maximal handgrip strength (recorded to the nearest tenth of a kg) was measured bilaterally with limbs unsupported and with the elbow in the extended position. Athletes were asked to squeeze the dynamometer as tight as possible. Each hand was measured twice, and the peak value obtained over the 2 trials was considered for the analysis [31].

### Statistical analysis

An inspection of the data revealed no missing values, nor were any univariate outliers found. A priori power analysis through G*Power (3.1.9.2) [32] was used to determine the required sample size considering the following input parameters: effect size d = 0.8; α = 0.05; statistical power = 0.9. The required minimum sample size was 58 (28 for each group), which was respected in the present study. Regarding to the multiple regression analysis, the required minimum sample size was considered based on the following input parameters: effect size f^2^ = 0.1; α = 0.05; statistical power = 0.9. was 88 (total sample), which was also respected.

Descriptive statistics (range, mean, standard error of the mean, and standard deviation) were calculated for the overall sample. Players were classified according to ethnicity, and body composition and maximal grip strength were compared between groups. Data normality was tested by the Kolmogorov-Smirnov test. A *t*-test for independent samples (variables with normal distribution) and Mann-Whitney (variables with non-normal distribution) were used to verify differences between ethnic groups (Black Africans and Caucasians). Additionally, an effect size analysis was used to determine the magnitude of the effect [33]. Cohen’s *d* was considered, whenever the *t*-test was used, and interpreted based on the following cut-off values: 0.0–2.0, trivial; 0.21–0.60, small; 0.61–1.20, moderate; 1.21–2.00, large; > 2.00, very large [34]. In cases where the Mann-Whitney test was used, η^2^ was obtained and the interpretation of the effect size was based on the following criteria: <0.01 no effect, 0.01–0.05 small effect, 0.06–0.13 moderate effect and ≥0.14 large effect [35].

In addition, a multiple linear regression analysis was performed to explore the predictors of the variables; BF% and Grip Strength. For BF% the independent variables included in the model were the following: Hours of training, Chronological Age (CA), Body Mass (BM), Height, Body Mass Index (BMI), Body Surface (BSA), Ethnicity and the circumferences (as previously described). Skinfolds were not included in the model to avoid multicollinearity situations. Regarding the Grip Strength, the independent variables included in the model were the following: Hours of training, Chronological Age (CA), Body Mass (BM), Height, Body Mass Index (BMI), Body Surface (BSA), Ethnicity, the 9 skinfolds and the 11 circumferences. The Stepwise method was applied in the multiple linear regression analysis. Ethnicity was considered as Black or Caucasian for comparison purposes in the model. The value of the ANOVA test was used to assess whether the model is a significant predictor. The adjusted R^2^ was used to evaluate the model’s power of explanation, and interpretation of R^2^ as an effect size measure was made based on the following criteria: 0.02–0.13 small, 0.13–0.26 medium, and >0.26 large effect size [36]. The assumptions underlying the use of the multiple linear regression model (normality, independence and homoscedasticity of the error and no situations of multicollinearity) were all met. The assumption of no extreme values was also verified.

Statistical analyses were performed using IBM Statistical Package for Social Science (SPSS) version 22.0 (SPSS, Inc., Chicago, Ilinois, USA) with the threshold for statistical significance set to *p* ≤ 0.05.

## Results

Descriptive statistics for variables measured regarding weekly training volumes, anthropometrics measures (Body mass, skinfolds, circumferences) and parameters extracted from the handgrip tests are presented in Table 1.

**Table 1:** Descriptive statistics combined for both ethnic groups.

Variable	Range (min-max)	Mean			(Kolmogorov-Smirnov)
		Value	Standard error	Standard deviation	p
Training, hours per week	7.50–12	10. 50	0.20	1,55	0.000
Chronological age, years	18–43	26.59	0.60	6.02	0.001
Body mass, kg	51.4–120.80	76.36	0.90	9.18	0.092
Height, cm	163.80–189.30	175.80	0.60	5.87	0.200
Body mass index, kg/m^2^	18.25–38.13	24.68	0.25	2.56	0.001
Body Surface Area, cm^2^	1.57–2.36	1.92	0.01	0.12	0.200
Abdominal skinfold, mm	5–52	16.20	0.81	8.33	0.002
Pectoral skinfold, mm	4–34	8.80	0.46	4.83	0.000
Front thigh skinfold, mm	5–36	12.45	0.55	5.80	0.000
Sum of skinfolds, mm	47–270	89.49	3.58	37.88	0.000
Body density, g/cm3	1.03–1.09	1.07	0.01	0.01	0.026
Body fat mass, %	3.86–31.93	10.82	0.48	5.07	0.021
Waist circumference, cm	71.5–111.5	84.81	0.58	5.98	0.020
Abdominal circumference, cm	68.2–118	82.79	0.65	6.74	0.003
Hip circumference, cm	78.6–117.8	99.07	0.53	5.55	0.042
Right thigh circumference, cm	50.5–75	60.98	0.43	4.47	0.200
Right mid-thigh circumference, cm	48.3–66	54.88	0.31	3.32	0.000
Right distal-tight circumference, cm	35–55	42.44	0.28	2.86	0.200
Right calf circumference, cm	32–44	36.96	0.22	2.30	0.200
Left thigh circumference, cm	50–73	60.06	0.40	4.15	0.200
Left mid-thigh circumference, cm	46.60–63	54.02	0.28	2.89	0.200
Left distal-tight circumference, cm	34.30–55.50	42.17	0.29	2.90	0.006
Left calf circumference, cm	31.30–46	36.76	0.23	2.42	0.071
Right grip strength, N	37.70–73.80	50.91	0.62	6.26	0.200
Left grip strength, N	37.30-79-91	50.27	0.63	6.23	0.082

Results presented as range, mean, standard error of the mean, 95% confidence limits of the mean, and standard deviation.

### Body composition and grip strength of elite rink hockey athletes

The results of Table 1 display the body composition traits and grip strength references of rink hockey athletes with a regular weekly training volume of 10.50 ± 1.55 hours. BM showed an average of 76.36 ± 9.18 kg, and an average of 175.8 ± 5.87 cm was assessed for height. BMI reveals a high limit for normal weight (24.68 ± 2.56 kg/m2). The summary of the 9 skinfolds (89.49 ± 37.88 cm) reflects a BF% of 10.82 ± 5.07%. The BSA is set at 1.92 ± 0.12 m^2^. Hand grip strength was tested, with 88% of the sample referenced as right-hand dominant and 12% left-hand dominant. For maximal RGST it was obtained at 50.91 ± 6.26 kg and 50.27 ± 6.23 kg for LGST.

Table 2 summarized the comparison between ethnicity group results. There were significant differences in several variables between players’ ethnicities. Caucasian athletes revealed significantly higher BM (77.97 ± 8.65 kg) than Black athletes (72.78 ± 9.47 kg). There were also significant differences in BMI and BSA between groups, 23.78 ± 3.08 kg/m^2^ and 1.87 ± 0.13 m^2^ for Black athletes, while 25.0 ± 2.19 kg/m^2^ and 1.94 ± 0.12 m^2^ for Caucasians athletes. Body density is significantly higher for Black athletes (1.076 ± 0.015) than for Caucasians (1.073 ± 0.009). Regarding the BF%, significantly higher results were observed for Caucasian rink hockey athletes (11.21 ± 4.13) than for Black athletes (9.94 ± 6.72).

**Table 2:** Descriptive statistics (mean ± standard deviation) by group and mean differences in anthropometry and strength variables.

Variable	Black African (*n* = 31)	Caucasian (*n* = 69)	Statistic’s value	*p-*value	Effect Size		
					Value	Qualitative	ICC (IC 95%)
Training, hours per week	8.7±1.30	11.3±0.93	149.50	<0.001^#2^	0.47^§2^	Large	
Chronological age, years	24.39±4.27	27.58±6.44	769.50	0.025^#2^	0.05^§2^	Small	
Body mass, kg	72.8±9.50	78.0±8.70	765	0.023^#2^	0.05^§2^	Small	
Height, cm	175.0±6.50	176.2±5.60	923	0.348^#1^	0.20^§1^	Trivial	-0.220–0.629
Body mass index, kg/m^2^	23.78±3.08	25.0±2.19	782	0.032^#2^	0.05^§2^	Small	
Body Surface Area, cm^2^	1.87±0.13	1.94±0.12	775.50	0.014^#1^	0.57^§1^	Small	0.137–0.999
Sum skinfold, mm	88.6±52.20	89.9±29.70	764.50	0.023^#2^	0.05^§2^	Small	
Body density, g/cm^3^	1.076±0.02	1.073±0.01	711.50	0.008^#2^	0.07^§2^	Moderate	
Body fat mass, %	9.9±6.70	11.2±4.10	711.50	0.008^#2^	0.07^§2^	Moderate	
Waist circumference, cm	81.27±6.45	86.40±5.05	445.50	<0.001^#2^	0.22^§2^	Large	
Abdominal circumference, cm	78.82±7.14	84.57±5.77	461.50	<0.001^#2^	0.21^§2^	Large	
Hip circumference, cm	96.54±6.85	100.20±4.46	643.50	0.001^#2^	0.10^§2^	Moderate	
Right thigh circumference, cm	58.91±5.40	61.90±3.66	636	0.008^#1^	0.70^§1^	Moderate	0.266–1.135
Right mid-thigh circumference, cm	54.57±4.37	55.02±2.76	931.50	0.303^#2^	0.01^§2^	Small	
Right distal-tight circumference, cm	41.65±2.95	42.79±2.77	897	0.198^#2^	0.02^§2^	Small	
Right calf circumference, cm	35.78±2.48	37.50±2.02	587	<0.001^#1^	0.79^§1^	Moderate	0.354–1.23
Left thigh circumference, cm	58.11±4.83	60.93±3.49	671	0.005^#1^	0.71^§1^	Moderate	0.279–1.149
Left mid-thigh circumference, cm	53.49±3.44	54.25±2.59	903	0.220^#1^	0.26^§1^	Small	-0.161–0.689
Left distal-tight circumference, cm	40.67±2.99	42.84±2.60	652.50	0.002^#2^	0.10^§2^	Moderate	
Left calf circumference, cm	35.43±2.56	37.36±2.11	554.50	<0.001^#2^	0.15^§2^	Large	
Right grip strength, kg	52.26±6.3	50.30±6.2	870.50	0.148^#1^	0.31^§1^	Small	-0.741–0.111
Left grip strength, kg	51.57±5.3	49.69±6.6	849.00	0.165^#1^	0.30^§1^	Small	-0.729–0.123

Note: #1- Independent samples t-test; #2- Mann-Whitney test; §1 –Cohen’s d; §2 - *η*^2^.

In regards to the circumference measurements, elite male Black rink hockey athletes had significantly lower values than Caucasian athletes in almost all variables, respectively; WC (-6%), ABC (-6.8%), HC (-3.7%), RTC (-4.8%), RCC (-4.6%), LTC (-4.6%), LDTC (-5.1%) and LCC (-5.2%).

There were no significant differences in grip strength between both ethnicities, however, mean values in black athletes were higher in both hands (52.26 ± 6.34 N; 51.57 ± 5.30 N), than in Caucasian athletes (50.30 ± 6.17 N; 49.69 ± 6.56 N).

Results of the multiple regression analysis in Tables 3 and 4 showed an overall significant model for BF% (p_ANOVA_ ≤ 0.05) and grip strength (p _ANOVA_ ≤ 0.05). Once the percentage of the dominant hand is near 90% of the right hand, the right grip strength was considered for the regression model. Moreover, there were no significant differences between right and left grip strength between the ethnic groups.

**Table 3:** Multiple linear regression model for BF% in elite male rink hockey athletes.

Model 1	Coefficients			ANOVA *p-*value	Adj. R^2^
	Estimates	p-value	CI_95%_		
Constant	-43.658	<0.001	(-53.124; -34.193)	<0.001	
Abdominal circumference	0.425	<0.001	(0.292;0.558)		0.701
Right thigh circumference	0.609	<0.001	(0.398;0.820)		
Right calf circumference	-0.440	0.001	(-0.797; -0.083)		
Ethnicity	-2.233	0.016	(-3.541; -0.925)		

**Table 4:** Multiple linear regression model for right grip strength in elite male rink hockey athletes.

Model 1	Coefficients			ANOVA *p-*value	Adj. R^2^
	Estimates	p-value	CI_95%_		
Constant	24.018	0.007	(6.684;41.352)	0.001	
Ethnicity	-3.335	0.013	(-5.939; -0.732)		0.131
Chronological Age	0.239	0.024	(0.033;0.446)		
Right distal-tight girth	0.538	0.014	(0.110;0.966)		

Regarding to the BF%, four predictors accounted for 70.1% (R^2^adj = 0.701) of its variance (ABC, RTC, RCC and ethnicity). According to the results, the following regression equations were obtained for BF%, for each ethnicity: Black Athletes = -43.658 + 0.425×ABC + 0.609×RTC—0.440×RCC; Caucasian Athletes = -45.891 + 0.425×ABC + 0.609×RTC—0.440×RCC.

Regarding the upper strength, three predictors accounted for 13,1% (R^2^adj = 0.131, medium) of the variance of RGST (Ethnicity, CA and RDTC). Despite the low value of R^2^, the analysis revealed a significant effect size, with a clear tendency of variance for the RGST. Based on this, the following regression equations were observed for RGST, for each ethnicity: Black Athletes = 24.018 + 0.239×CA + 0.538×RDTC; Caucasian Athletes = 20.683 + 0.239×CA + 0.538×RDTC.

## Discussion

The aim of the present research was to characterize the body composition and grip strength of elite male rink hockey players and to underline the possible relationships between ethnicity, body composition and grip strength. In line with our expectations, results revealed that there are significant differences between body composition and grip strength Black Africans and Caucasian elite rink hockey players.

To the best of our knowledge, this is the first study with a relatively large sample size (n = 100) of male rink hockey athletes comprising elite international Black African and Caucasian players. There are currently a limited number of studies published regarding body composition and grip strength, as well as other conditioning and physiological characterizations, of such athletes. The existing research is mainly focused on children and adolescent players [37]. Furthermore, research with elite adult athletes was assessed with a small sample size [37] and only with Caucasians. We believe that the standards presented in the current study may provide a target that coaches, physical conditioning and strength coaches, researchers, and clinicians can use to evaluate elite rink hockey players during an entire season. The assessment of body composition analysis seems to be an important monitoring tool which complements the understanding of the athlete’s performance and cardiac adaptation [17]. On the other hand, grip strength also plays a role in performance, strength, and injury prevention [17, 38]. There is a limited number of studies regarding the general characterization of rink hockey demands [37]. Moreover, the understanding of physical and physiological requirements is essential for game performance. Additionally, it is also crucial to complement athletes’ fitness traits and game characterization [39].

On the descriptive values of the sample, our results demonstrate that some body composition indicators, such as height, weight, and BF%, were lower than previously reported in research with 41 Spanish elite male rink hockey players [40]. However, in our study, BMI was similar to values reported in previous research [40]. Our results are also in line with the results of Santos et al. [41] with 49 rink hockey athletes. However, the overall value of BF% was slightly lower (-1%). This difference may be suggested by the technique of assessment. Dual-energy X-ray absorptiometry (DXA) is a convenient and useful tool for body composition valuation [42], due to the speed and reliability of analysis and at the same time, due to its minimal measurement discrepancy in the water factor [43,44]. Regarding the summary of skinfolds (SUMSK) and DXA, the research of Santos et al. [41] suggests an alignment between both methods. As reported in other sports, these results indicate the existence of an anthropometric identity of elite rink hockey, which may be considered as a reference to achieving greater performance [6–8].

In research involving young men, maximal grip strength and BF% were higher than that registered in this study [45]. Similar BF% was also observed in active young men with regular practice of cycling [46]. Moreover, elite rink hockey players seem to have lower BF% when compared with elite ice hockey players [47]. Besides suggestions of similarities in internal and external load between both modalities, these differences may be based on the evolutionary adaptation of elite ice hockey athletes, as reported by Ashley et al. [47]. The author suggests that the increase in physical size and fitness parameters of National Hockey League (NHL) players, such as stature, body mass, aerobic fitness, peak power output, and grip strength may be a result of the professional competition and its rigorous schedule which demands greater physical and physiological requirements on players [47–49]. On the other hand, when compared with other hockey modalities, the size of the rink hockey field may influence the volume of requirements of short and long-duration efforts, its incomplete recovery periods and consequently, its metabolic demands [1,3]. Additionally, the lack of information regarding external load during competition may impact a greater interpretation of players’ adaptations to the game demands.

Strength training is an essential part of a physical training program [50]. Thus, the measurement of grip strength can be an important part of muscular strength assessment by providing a fast estimation of athletes’ upper body strength [17,50]. Besides its relevant insights into an individual´s bone density [13,16] and functional status [14], grip strength is mandatory in most hockey-specific movements with players continually performing complex skills while gripping a stick [5]. It is therefore probable that rink-hockey players may have higher grip strength, given these neuromuscular adaptations from training [17]. In fact, our results reveal that elite hockey players have noticeable higher values than active adults and amateur soccer players [51]. However, when compared with elite ice hockey athletes, the values of elite rink hockey grip strength are lower and differences within both hands are greatly evident [18]. Research regarding the association of maximal strength and muscular endurance test scores with cardiorespiratory fitness and body composition of 846 young men, revealed greater grip strength and BF% than our elite rink hockey athletes [19]. Grater grip strength values were also observed in other elite athletes such as in handball, water polo, tennis, or gymnastics [52]. These differences may open new perspectives concerning the need of understanding the importance of strength training for elite rink hockey athletes and its implication on their performance. Several studies associate body composition and strength scores with cardiorespiratory fitness. In our research that association was not possible. Additionally, the lack of information regarding the training and competition loads of the teams who participated in this research may have impacted the discussion of the results of the measured variables. It has been already suggested that there is a need to clarify the elite athletes’ traits and game demands to develop new evaluation and monitoring tools which will improve rink hockey training environments and consequently performance levels [37].

Biological differences exist in the body composition of Black and Caucasians [21]. The differences and similarities which have been investigated between the two ethnicities are related to fat-free body mass and fat patterning body dimensions, and proportions [9,21]. In our research, several differences were reported in the body composition variables of the elite rink hockey athletes. In all variables, values were significantly lower in black athletes except for body density. Therefore, we believe that the results of our research are line with previous studies. In general, elite male Black rink hockey athletes have a greater bone mineral density and body protein content than their Caucasian counterparts, resulting in a greater fat-free body density [9,21]. For example, Vickey et al. [53] reported that the mean value of measured body density was significantly higher in Black athletes than in Caucasians. Regarding athletes, in research with Olympic athletes, Malina et al. [54] reported that BF% is influenced primarily by sports and training, whereas fat patterning is more dependent on biological factors. In this study, Black athletes have significantly lower weekly training hours than their Caucasian peers, even though, significantly lower BF% and SUMSK, and significantly higher BD have been reported. Consequently, we suggest that these differences in elite rink hockey athletes are greater related to genetic factors than within training volumes. In the same line, there are no significant differences in both grip strength, as Black athletes reveal higher mean values than Caucasians. Regarding this result, it is not possible to underline if the volume and/or typification of strength work in both championships impact the existence of significant statistical differences.

In general, our results were in accordance with previous studies. The literature shows reliable differences in fat patterning between Blacks and Caucasians [9,21]. Additionally, these differences in fat deposition could produce systematic errors in the estimates of BF% using field methods that assume a consistent fat patterning between the two ethnicities [21]. Following that most equations that predict relative body fat were derived from predominantly Caucasian populations, some researchers emphasize that generalized equations need to be cross validated in Black populations [21,55].

Our results of the multiple regression analysis showed an overall significant model for BF% and grip strength for each ethnicity. ABC, RTC, RCC and ethnicity demonstrated a significant interaction effect on BF%. Therefore, it seems that between other variables, the effect of ethnicity was presented on both estimated equations of BF% and grip strength for rink hockey athletes. Regarding the other predictors of BF%, abdominal circumference results were in line with the literature. Several researchers have shown lower abdominal, abdominal subcutaneous, and visceral fat during regular and progressive intense training such as in rink hockey in comparison with people without intense practice [17,40,56]. In previous studies, prediction models used the circumference of arms, thigh, and calf, skinfolds, and body height as independent variables [57]. These anthropometric variables derived from body mass are related to fat mass and fat-free mass in athletes [58]. In this study, right thigh and right calf circumferences demonstrated a significant interaction effect on the BF% of elite male rink hockey athletes. It has been suggested that proportions of segment fat mass and fat-free mass within the whole body for athletes are event specific [59,60]. Therefore, we believe that such predictors may be justified by the existence of specific movements and energy requirements or even habitual training adaptations of this sport [57]. On the other hand, the lack of information regarding the external load may be a limitation in better explaining these results. Further studies should be developed to extend this research to include more conditioning data and the understanding of how external game demands may be related or influenced by an athlete’s conditioning profile.

Regarding the upper strength, three predictors showed an overall significant model for the variance of right grip strength. Ethnicity, age, and right distal thigh circumference were included in the model. As in BF%, ethnicity seems to be a predictor of this variable. On the other hand, as reported in research with ice hockey athletes, grip strength increases with age [5]. Therefore, regarding the neuromuscular adaptation of griping the stick [17] like in ice hockey [5] and according to our multiple regression, we believe that the importance of age predicting grip strength is in accordance with this previous result. Finally, in line with the third predictor (RDTC) of grip strength, the literature shows a previous relationship between the recovery after total knee arthroplasty and hand-grip strength [61]. However, we suggest that this variable predictor may be influenced by players’ adaptations to the continuous high intense intermittent game requirements such as accelerations, decelerations, and multidirectional speed [37]. As previously mentioned, further research should be taken regarding the characterizations of training and competition external load, and consequently athletes’ adaptations.

### Practical applications

Body composition and grip strength assessments may be important for coaches, strength and conditioning trainers and clinicians working with elite rink-hockey players, to facilitate comparison of performance and players’ traits, allowing to establish a reference standard. We believe that such information is useful to assess and monitor changes in standards of performance, injury management, and strength/skill developments in elite rink-hockey players. Regarding the training monitoring, we believe that these results may establish reference information that might help to shed a light on elite rink hockey athletes’ performance and training adaptations. Consequently, the need to understand athletes’ traits may open a new era in rink hockey by improving training methods and increasing players’ performance, as has been observed in other sports modalities and their athletes since they have taken part in intended and longitudinal research [6,43]. Consequently, with these results, we hope to bring new insight into information and technical monitoring regarding elite rink hockey traits and performance.

## Conclusions

Rink hockey at an elite level may lead to reduced BF% and greater handgrip strength. Abdominal circumference, right circumference, right calf circumference and ethnicity seem to demonstrate a significant interaction effect on BF%. Additionally, ethnicity, chronological age, and right distal thigh circumference constraints the variance of handgrip strength in elite male rink hockey athletes.

## Supporting information

S1 File Full dataset. (XLSX)
